# Supplementary material for: Yiai Fuzheng decoction inhibits triple-negative breast cancer by remodeling the immune microenvironment
Source: Front Immunol. 2025 Sep 30;16:1615631. doi: 10.3389/fimmu.2025.1615631 (PMC12518410; doi:10.3389/fimmu.2025.1615631)
Supplement: Supplementary file 2 [file DataSheet1.zip › Supplementary File 2/Treatment/D_vs_H/07_Pathway_Analysis/Pathway_Analysis/Sig_Pathways_ID/beta-Alanine metabolism.pdf]

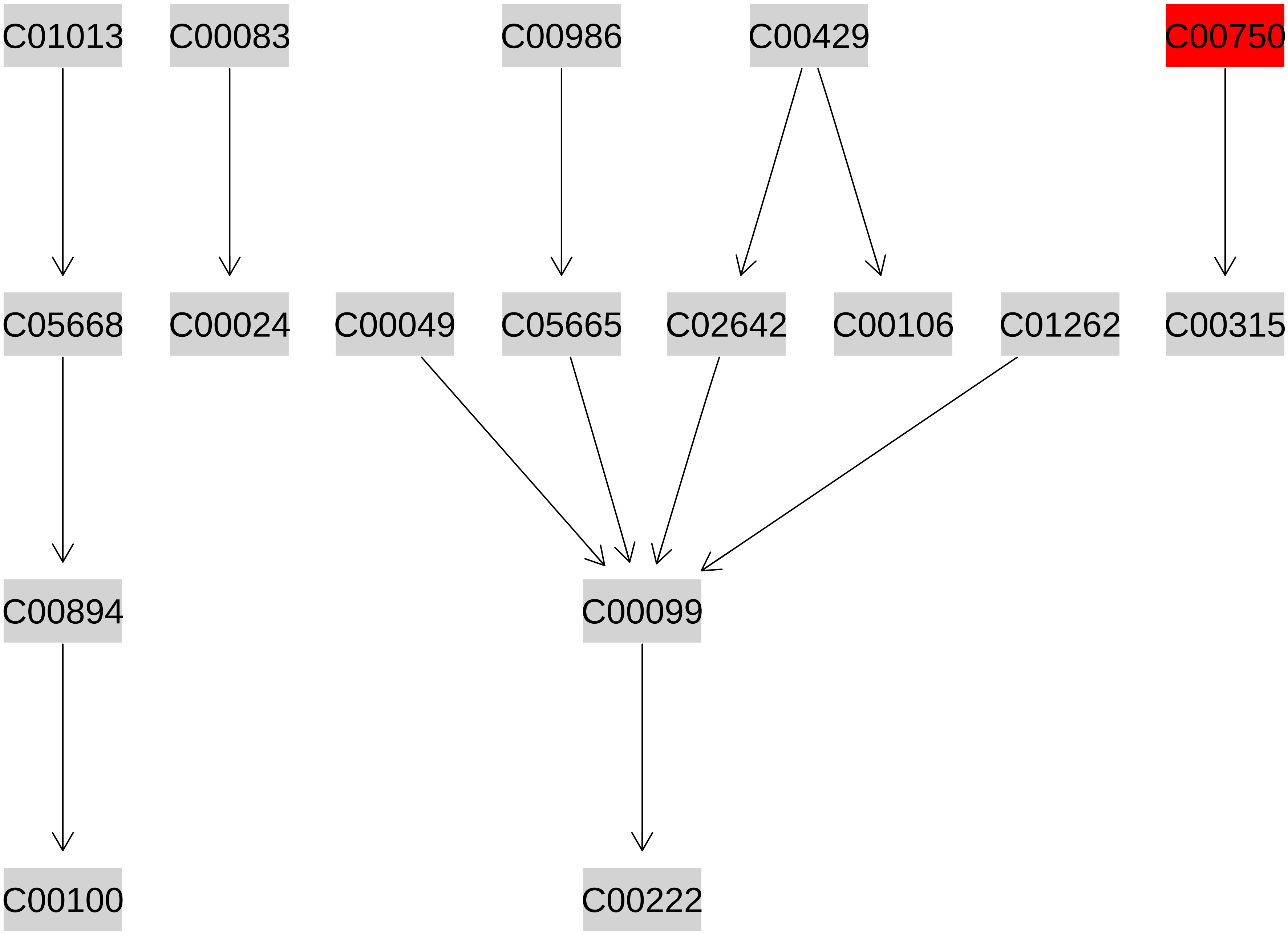
C01013
C00083
C00986
C00429
C00750
C05668
C00024
C00049
C05665
C02642
C00106
C01262
C00315
C00894
C00099
C00100
C00222
